# The risk of subsequent invasive melanoma after a primary in situ or invasive melanoma in a high incidence country (New Zealand)

**DOI:** 10.1002/ski2.116

**Published:** 2022-05-13

**Authors:** Thu Thu Win Myint, Vanessa Selak, Mark Elwood

**Affiliations:** ^1^ Department of Biostatistics and Epidemiology University of Auckland Auckland New Zealand

## Abstract

**Background:**

Patients with invasive melanoma are at increased risk of developing subsequent invasive melanoma, but the risks for those with primary in situ melanoma are unclear.

**Objectives:**

To assess and compare the cumulative risk of subsequent invasive melanoma after primary invasive or in situ melanoma. To estimate the standardized incidence ratio (SIR) of subsequent invasive melanoma compared to population incidence in both cohorts.

**Methods:**

Patients with a first diagnosis of melanoma (invasive or in situ) between 2001 and 2017 were identified from the New Zealand national cancer registry, and any subsequent invasive melanoma during follow‐up to the end of 2017 identified. Cumulative risk of subsequent invasive melanoma was estimated by Kaplan–Meier analysis separately for primary invasive and in situ cohorts. Risk of subsequent invasive melanoma was assessed using Cox proportional hazard models. SIR was assessed, allowing for age, sex, ethnicity, year of diagnosis and follow up time.

**Results:**

Among 33 284 primary invasive and 27 978 primary in situ melanoma patients, median follow up time was 5.5 and 5.7 years, respectively. A subsequent invasive melanoma developed in 1777 (5%) of the invasive and 1469 (5%) of the in situ cohort, with the same median interval (2.5 years) from initial to first subsequent lesion in both cohorts. The cumulative incidence of subsequent invasive melanoma at 5 years was similar in the two cohorts (invasive 4.2%, in situ 3.8%); the cumulative incidence increased linearly over time in both cohorts. The risk of subsequent invasive melanoma was marginally higher for primary invasive compared to in situ melanoma after adjustment for age, sex, ethnicity and body site of the initial lesion (hazard ratio 1.11, 95% CI 1.02–1.21). Compared to population incidence, the SIR of invasive melanoma was 4.6 (95% CI 4.3–4.9) for the primary invasive and 4 (95% CI 3.7–4.2) for the primary in situ melanoma cohorts.

**Conclusions:**

The risk of subsequent invasive melanoma is similar whether patients present with in situ or invasive melanoma. Thus follow‐up surveillance for new lesions should be similar, although patients with invasive melanoma require more surveillance for recurrence.

## Graphical abstract

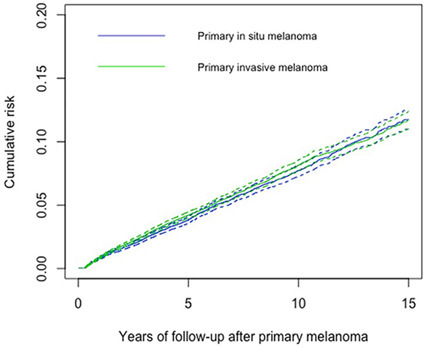

1

**What is already known about this topic?**

- Patients with primary invasive melanoma are at increased risk of developing subsequent invasive melanoma but it is unclear how this risk compares with that for patients with primary in situ melanoma.

**What does this study add?**

- The cumulative risk of subsequent invasive melanoma at 5 years' follow‐up was similar among primary invasive (4.2%) and in situ (3.8%) melanoma patients.
- The standardized incidence ratio (SIR) of subsequent invasive melanoma was increased and of similar magnitude, with SIRs of 4.6 (95% CI, 4.3–4.9) for primary invasive and 4 (95% CI, 3.7–4.2) for in situ melanoma patients compared to population incidence.
- Patients with primary in situ (as well as invasive) melanoma should be considered for follow‐up surveillance for new lesions.

## 2 INTRODUCTION

Melanoma is the 19th most common cancer globally^1^ and the third most common cancer in New Zealand.^2^ The incidence of melanoma has been increasing worldwide over the past 50 years, especially in fair‐skinned populations and people living in areas of lower latitude.^3^ New Zealand and Australia experienced the greatest burden of melanoma followed by North America and European countries in 2013^4^ and 2015.^5^

Melanoma is diagnosed as either an in situ or invasive lesion. It has been suggested that simple excision of in situ lesions might prevent progression to invasive lesions and metastasis.^6^ The annual incidence of in situ melanoma has increased at a faster rate to that of invasive melanoma in Australia,^7^, ^8^, ^9^, ^10^ New Zealand,^11^, ^12^ Italy,^13^, ^14^, ^15^ Spain^16^ and the United States.^17^, ^18^, ^19^ However, the relationship between in situ and invasive melanoma remains unclear^11^ even though many previous studies have compared their epidemiological characteristics^7^, ^8^, ^9^, ^10^, ^11^, ^12^, ^13^, ^14^, ^15^, ^16^, ^17^, ^18^, ^19^, ^20^, ^21^, ^22^, ^23^, ^24^, ^25^, ^26^, ^27^, ^28^, ^29^ or subsequent risk of melanoma.^27^, ^30^, ^31^, ^32^, ^33^, ^34^ The risk of subsequent melanoma of either type was found to be higher among the patients with primary in situ melanoma when compared to those with primary invasive melanoma for some studies,^30^, ^31^, ^32^, ^33^, ^34^ whereas a Queensland study^27^ found that the risk of subsequent invasive melanoma was greater for patients with primary invasive melanoma compared to those with primary in situ melanoma. The aim of this study was to determine whether the incidence of subsequent invasive melanoma differed according to primary melanoma type (in situ or invasive), and whether the incidence varied by key characteristics (sex, age groups, ethnicity and body site of primary melanoma), using national population‐based cohorts.

## 3 PATIENTS AND METHODS

Patients with their first diagnosed melanoma (either in situ or invasive) diagnosed from 1 January 2001 to 31 December 2017 were identified from the New Zealand Cancer Registry (NZCR). NZCR^35^ is a population‐based, legislatively mandated, cancer registry in New Zealand which receives all pathology reports of malignant tumours. In situ melanoma pathology reports are received and coded in the same way as invasive reports, although data on in situ lesions are not routinely reported. Data were linked to subsequent melanoma diagnoses (also from the NZCR) and/or death (from national mortality data collection) during follow‐up (up to 31 December 2017), and de‐identified data then used for analysis. Follow up time was from 3 months after the initial diagnosis date, as patients with synchronous diagnosis within 90 days were excluded for follow‐up analysis. A few previous studies of follow up made a similar adjustment.^33^, ^36^ The restriction was applied because synchronous diagnosis may have been influenced by the health care attention the patient received, or may be related to the primary lesion. An event was categorized as ‘1’ for subsequent diagnosis of invasive melanoma after primary melanoma (either invasive or in situ) whereas censoring was categorized as ‘0’ at the time of death or at the end of study period. Thirty‐four patients whose sex and ethnicity were unknown, or whose domicile code was invalid were excluded from the study.

The cumulative incidence of subsequent primary invasive melanoma after diagnosis with primary in situ or invasive melanoma was assessed by non‐parametric Kaplan–Meier analysis at 5, 10 and 15 years of follow‐up time by key characteristics such as sex, age group, ethnicity and body site, separately by primary melanoma type (invasive or in situ).

The cumulative risk differences were tested by the log‐rank test within key characteristics of each primary melanoma type and between the two cohorts of primary melanoma for each key characteristic. The risks of subsequent invasive melanoma among the primary invasive and in situ melanoma cohorts were compared according to key characteristics (univariate and multivariate) in Cox proportional hazard models. Univariate models assessed the impact of covariates, and multivariate model measured the impact of other covariates when the risk was estimated for the one factor after adjustment.^37^ The proportionality assumption in each model was assessed by using the global Schoenfeld test^38^ and plotting log[−log(survival)] versus log(time).

The incidence of subsequent invasive melanoma relative to population incidence was estimated by standardized incidence ratio (SIR). This analysis was done, separately for each sex, for patients aged between 30 and 74 years in the two largest groups in New Zealand statistics, the ‘Other’ (predominantly New Zealand European) ethnic group and the Māori ethnic group. A more limited age range was used because melanoma in younger and older subjects may differ both in biology and diagnosis. The observed number of subsequent invasive melanoma from each cohort of primary melanoma type was compared with the expected number of subsequent invasive melanoma, taking into account year of diagnosis, exact follow up duration, sex, age, ethnicity and body site of primary melanoma. A detailed explanation of the calculation is provided in Appendix S1 and Table S1.

## 4 RESULTS

This population‐based cohort study included 61 262 eligible patients with a first diagnosed invasive or in situ melanoma from 1 January 2001 to 31 December 2017; 33 284 (54%) patients made up the cohort with an initial primary invasive melanoma and 27 978 (46%) patients formed the cohort with initial primary in situ melanoma. Median follow up time was 5.5 years (interquartile range, 2.1–10 years) for invasive cohort and 5.7 years (interquartile range, 2.4–9.9 years) for in situ cohort with the same interval of 2.5 median years between initial and subsequent diagnoses. The same proportion (5%) of subsequent invasive melanoma developed with 1777 patients in primary invasive melanoma and 1469 patients in primary in situ melanoma, excluding the synchronous diagnosis of melanoma within 90 days. There were 1075 cases of subsequent invasive cases (550 cases from invasive cohort, 525 cases from in situ cohort) developed within 90 days of their first melanomas.

Table 1 describes the key characteristics of patients in the primary invasive and in situ melanoma cohorts. Patients with invasive melanoma were slightly younger at diagnosis than those with in situ melanoma (median age 63 vs. 64 years, *p* < 0.001 by the Wilcoxon–Mann–Whitney test), though this difference is unlikely to be clinically significant. Majority of primary melanoma developed in 60–69 years age groups and few lesions were found in extreme age groups. Males had a higher proportion of both types of primary melanoma than females. Most melanomas were found in European/Other ethnic group. Trunk was the most common region for both types of primary melanomas followed by upper and lower limbs in primary invasive, and face and upper limbs in primary in situ cohort.

**TABLE 1:** Key characteristics of patients diagnosed with either primary invasive or in situ melanoma

Key characteristics	Primary invasive melanoma, *n* (%)	Primary in situ melanoma, *n* (%)
	33 284	27 978
Age at diagnosis		
Mean (SD)	62.2 (16)	63.1 (14.3)
Median (IQR)	63 (51–74)	64 (54–74)
Age groups		
10–19	121 (0%)	58 (0%)
20–29	759 (2%)	390 (1%)
30–39	2117 (6%)	1243 (4%)
40–49	4324 (13%)	3181 (11%)
50–59	6551 (20%)	5773 (21%)
60–69	7608 (23%)	7504 (27%)
70–79	6734 (20%)	6212 (22%)
80–89	4234 (13%)	3194 (11%)
90+	834 (3%)	423 (2%)
Sex		
Female	15 985 (48%)	13 563 (49%)
Male	17 299 (52%)	14 415 (51%)
Ethnicity		
Māori	633 (2%)	447 (2%)
Pacific	104 (0%)	48 (0%)
Asian	64 (0%)	39 (0%)
European/Other	32 483 (98%)	27 444 (98%)
Body sites of first melanoma		
Face	3353 (10%)	6962 (25%)
Scalp and neck	2034 (6%)	1721 (6%)
Trunk	10 183 (31%)	7952 (28%)
Upper limbs	7576 (23%)	6345 (23%)
Lower limbs	8605 (26%)	4855 (17%)
Other area	1525 (5%)	123 (0%)
Overlapping area	8 (0%)	20 (0%)

### 4.1 Cumulative incidence of subsequent invasive melanoma

The overall cumulative risks of subsequent invasive melanoma were 4% at 5 years, 8% at 10 years and 11.7% at 15 years of follow‐up time. Table 2 describes and compares the cumulative incidence of subsequent invasive melanoma between a cohort of patients with first invasive and a cohort of patients with in situ melanoma, and between each key patient characteristic (age, sex, ethnicity and body site) for each cohort. The risks between the two cohorts were closely similar with 4.2% and 8.2% for invasive cohort and 3.8% and 7.7% for in situ cohort at 5 and 10 years of follow‐up. No statistical difference in risks was noted between the two cohorts (*p* = 0.508). The risks were significantly different within sex, ethnic groups, age groups and body sites of primary melanoma for each cohort. The risks were higher in males, in the NZ European population, in older age groups and in the scalp and neck region; the associations with these factors were similar in the two cohorts.

**TABLE 2:** The cumulative risk of subsequent invasive melanoma at 5, 10 and 15 years of follow‐up and a long‐rank test with *p* value

Key characteristics	Number of subsequent invasive melanoma	Cumulative risk (%)			*p*‐Value
		5‐year	10‐year	15‐year	
Primary melanoma type					
Invasive	1469	4.2	8.2	11.7	0.508
In situ	1777	3.8	7.7	11.7	
Primary invasive melanoma					
Sex					
Female	699	3.3	6.4	8.8	0.000
Male	1078	5.1	10.0	15.0	
Ethnicity					
European/Other	1739	4.3	8.3	12.0	0.004
Māori	17	2.2	5.0	5.7	
Age groups					
20–29	12	0.8	1.7	2.5	0.000
30–39	67	1.7	3.6	5.6	
40–49	161	2.5	4.7	6.5	
50–59	355	3.6	7.2	11.5	
60–69	456	4.3	9.2	14.2	
70–79	493	6.4	12.8	18.6	
80–89	203	5.9	11.8	15.8	
Body sites of primary melanoma					
Face	200	5.2	9.9	12.7	0.000
Scalp and neck	620	5.1	10.5	14.9	
Trunk	386	4.7	9.0	12.9	
Upper limbs	408	3.6	7.8	12.0	
Lower limbs	126	3.6	6.5	9.5	
Primary in situ melanoma					
Sex					
Female	596	3.0	6.1	9.3	0.000
Male	873	4.6	9.3	14.3	
Ethnicity					
European/Other	1450	3.9	7.9	12.0	0.006
Māori	11	2.5	3.6	3.6	
Age groups					
20–29	5	0.9	1.8	1.8	0.000
30–39	31	1.9	2.7	4.9	
40–49	95	1.5	3.9	5.9	
50–59	247	2.4	5.8	8.9	
60–69	447	4.3	8.3	14.4	
70–79	436	5.2	11.7	17.7	
80–89	191	6.5	12.4	16.5	
Body sites of first melanoma					
Face	421	4.3	8.5	12.6	0.000
Scalp and neck	390	6.0	11.0	16.7	
Trunk	316	3.5	7.8	12.6	
Upper limbs	216	3.6	7.5	11.2	
Lower limbs	119	3.2	6.0	8.8	

The cumulative incidence of subsequent invasive melanoma was found to increase constantly, with no evidence of a decrease or an increase over time. The risks, and the patterns of risk for the two cohorts were very similar (Figure 1a). This constant linear increase in risks was noted in both sexes (Figure 1b), European population (Figure 1c), in trunk, upper limbs and lower limbs of primary melanoma sites (Figure 1d,e) and patients aged between 50 and 79 years (Figure 1f,g). The risk patterns were found to be similar between invasive and in situ melanoma across sexes, ethnic groups, body sites and age groups except in face, scalp and neck and in extreme of ages (less than 30 and more the 80 years; Figure 1a–g).

FIGURE 1Cumulative risk of subsequent invasive melanoma at 5, 10 and 15 years of follow‐up since after their first diagnosis of primary melanoma (a) by primary melanoma type (b) by sex and primary melanoma type (c) by ethnic group and primary melanoma type (d) by body sites of primary invasive melanoma (e) by body sites of primary in situ melanoma (f) by age groups of invasive melanoma (g) by age groups of in situ melanoma

**Figure:**
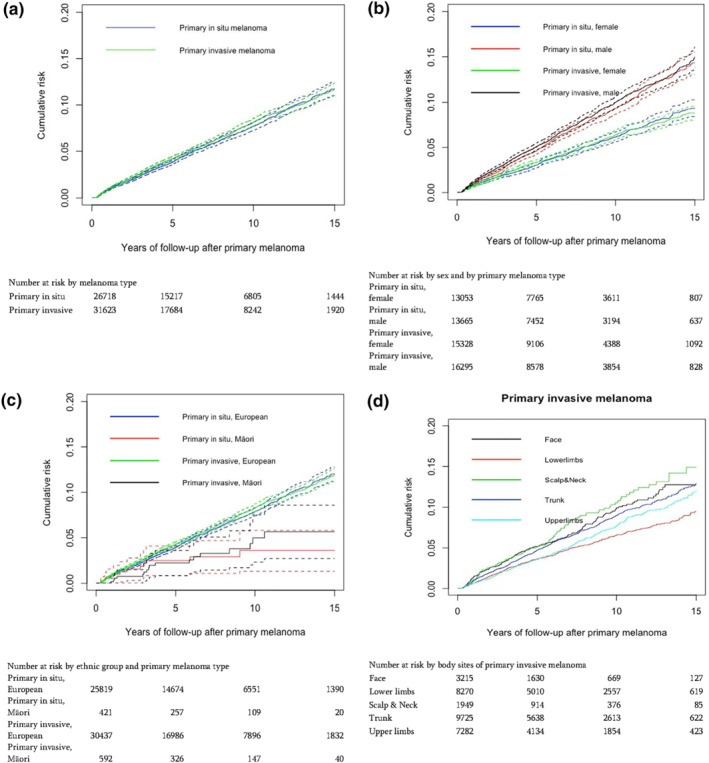

**Figure:**
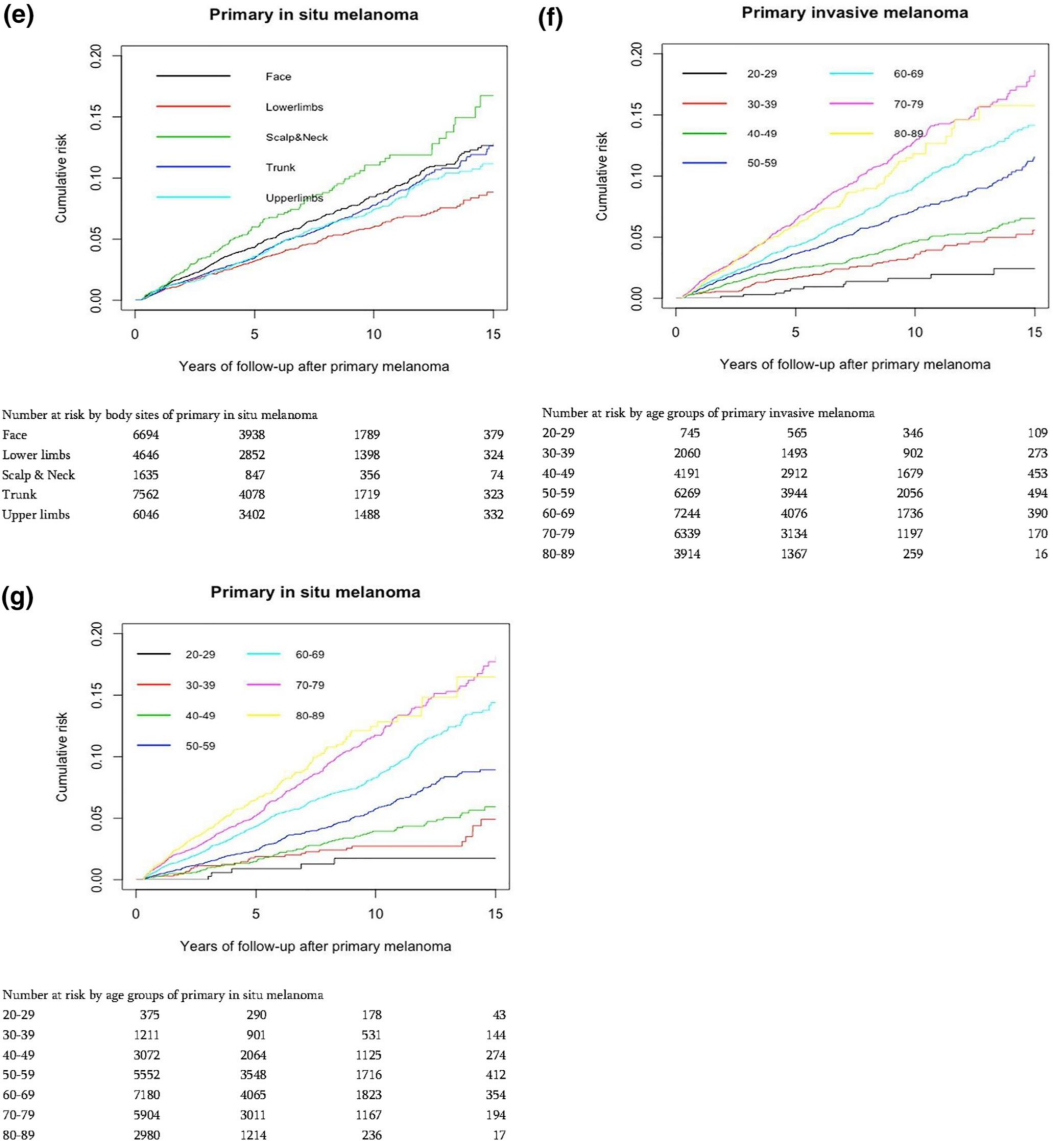

### 4.2 Comparison between invasive and in situ cohorts

The risk difference for subsequent invasive melanoma was compared between primary invasive and in situ melanoma cohorts (in situ as a reference) and for male, female, European, Māori, each body site and each age group in terms of univariate analysis (Table 3). The subsequent risk of invasive melanoma for patients with primary invasive melanoma was 7% higher than those with primary in situ melanoma in an unadjusted analysis but the difference was not statistically significant (95% CI 0.98–1.16, Table 3). No differences in unadjusted hazard risk between invasive and in situ cohorts were observed in more detailed analysis by sub‐categories except in the 50–59‐year‐old age group; the risk was 27% higher for invasive melanoma than in situ lesions among this age group.

**TABLE 3:** Risk difference for subsequent invasive melanoma between primary invasive and in situ melanoma cohort (in situ as a reference) by Log‐rank test and the unadjusted hazard ratios (HRs) for the cohort of primary invasive melanoma compared to the cohort of in situ melanoma by each sub‐category of key characteristics

Key characteristics	Log‐rank test (*p* value) for differences between primary invasive and in situ melanoma	Unadjusted HRs (95% CIs) for primary invasive compared to in situ melanoma
Primary melanoma type	0.118	1.07 (0.98–1.16)
Sex		
Female	0.170	1.10 (0.96–1.25)
Male	0.382	1.05 (0.94–1.17)
Ethnicity		
European/Other	0.144	1.06 (0.98–1.16)
Māori	0.138	2.17 (0.78–6.02)
Body sites of first melanoma		
Face	0.092	1.22 (0.97–1.54)
Scalp and neck	0.979	1.00 (0.74–1.36)
Trunk	0.103	1.13 (0.98–1.30)
Upper limbs	0.808	0.98 (0.82–1.16)
Lower limbs	0.338	1.10 (0.91–1.32)
Age groups		
30–39	0.367	1.22 (0.79–1.87)
40–49	0.225	1.17 (0.91–1.51)
50–59	0.005	1.27 (1.08–1.49)
60–69	0.812	1.02 (0.89–1.16)
70–79	0.095	1.17 (0.97–1.40)

### 4.3 Factors related to risks of melanoma—multivariate analysis

The adjusted hazard ratios (HRs) for subsequent invasive melanoma were compared between patients with a first invasive and a first in situ melanoma (Table 4). The risk of subsequent invasive melanoma was 11% (95% CI 1.02–1.21) higher in primary invasive cohort compared to in situ cohort after adjustment for age, sex, ethnicity and body site. The risk increased by 4% with each increase in age at diagnosis (in years), and males had a 38% higher risk than females for total primary melanoma and also for the two cohorts separately. Compared to the European population, Māori experienced 47% lower risk of subsequent invasive melanoma among total primary melanoma and the risk was reduced by 67% in the primary in situ cohort. Scalp and neck was noted as the body site with highest risk for subsequent invasive melanoma compared to face for total primary melanoma cohort and the two cohorts.

**TABLE 4:** Adjusted hazard ratios (HRs) with 95% CI for subsequent invasive melanoma between patients with primary invasive and in situ melanoma

Key characteristics	Total primary melanoma cohort (in situ + invasive)	Primary in situ melanoma	Primary invasive melanoma
Primary melanoma type			
Primary in situ melanoma	1	‐	‐
Primary invasive melanoma	1.11 (1.02–1.21)	‐	‐
Age, per year	1.04 (1.03–1.04)	1.04 (1.03–1.05)	1.03 (1.03–1.04)
Sex			
Female	1	1	1
Male	1.38 (1.27–1.52)	1.43 (1.26–1.63)	1.34 (1.19–1.51)
Ethnicity			
European/Other	1	1	1
Māori	0.53 (0.34–0.84)	0.33 (0.14–0.80)	0.69 (0.40–1.16)
Body sites of first melanoma			
Face	1	1	1
Scalp and neck	1.50 (1.24–1.81)	1.58 (1.23–2.04)	1.35 (1.02–1.79)
Trunk	1.19 (1.04–1.36)	1.22 (1.03–1.45)	1.10 (0.89–1.37)
Upper limbs	1.13 (0.98–1.30)	1.26 (1.05–1.51)	0.98 (0.78–1.23)
Lower limbs	1.07 (0.92–1.24)	1.18 (0.96–1.45)	0.94 (0.75–1.18)

*Note*: Column 3 compares the risk of subsequent invasive melanoma by each key patient characteristic (age, sex, ethnicity and body site) after adjustment for covariates among patients with primary in situ melanoma. This is the same for column 4, for primary invasive melanoma cohort.

### 4.4 Risk of subsequent invasive melanoma relative to population incidence

The risk of subsequent invasive melanoma was estimated by SIR (ratio of observed cases to expected cases in the general population) for those aged between 30 and 74 years, restricted to the Māori and European population (Table 5). The subsequent invasive melanomas were more likely to develop in primary invasive cohort compared to the primary in situ cohort, which was statistically significant (incidence rate ratio (IRR) 1.2, 95% CI 1.1–1.3). This pattern was similar in both sexes, Māori and European population, those aged 50–59 years, and in face and upper limbs of primary melanoma in further detailed analysis by sub‐categories (Table S2).

**TABLE 5:** SIRs and IRRs of subsequent invasive melanoma among primary invasive and in situ melanoma cohort by sex and ethnicity, and by age groups

Key characteristics	Observed (O)	Expected (E)	Standardized incidence ratio, SIR (95% CIs)	Incidence rate ratio, IRR (95% CIs)
Primary melanoma type				
Primary invasive	1273	277.2	4.6 (4.3–4.9)	1.2 (1.1–1.3)
Primary in situ	1032	261.2	4.0 (3.7–4.2)	
Primary invasive melanoma				
Sex				
Male	751	168.3	4.5 (4.1–4.8)	0.9 (0.8–1.0)
Female	522	108.9	4.8 (4.4–5.2)	
Ethnicity				
Māori	14	1.4	9.9 (5.4–16.7)	2.2 (1.0–3.3)
European/Other	1259	275.8	4.6 (4.3–4.8)	
Age groups				
30–39	67	8.7	7.7 (6.0–9.8)	1.9 (1.4–2.4)
40–49	157	27.4	5.7 (4.9–6.7)	1.4 (1.2–1.7)
50–59	352	62.0	5.7 (5.1–6.3)	1.4 (1.2–1.6)
60–69	442	108.2	4.1 (3.7–4.5)	Reference
70–79	255	71.0	3.6 (3.2–4.1)	0.9 (0.8–1.0)
Body sites of first melanoma				
Face	105	25.1	4.2 (3.4–5.1)	Reference
Scalp and neck	315	72.4	4.4 (3.9–4.9)	1.0 (0.8–1.3)
Trunk	88	15.2	5.8 (4.6–7.1)	1.4 (1.0–1.8)
Upper limbs	477	99.3	4.8 (4.4–5.3)	1.1 (0.9–1.4)
Lower limbs	288	65.2	4.4 (3.9–5.0)	1.1 (0.9–1.3)
Primary in situ melanoma				
Sex				
Male	616	161.1	3.8 (3.5–4.1)	0.9 (0.8–1.0)
Female	416	100.1	4.2 (3.8–4.6)	
Ethnicity				
Māori	5	1.3	3.9 (1.2–9.3)	1.0 (0.1–1.9)
European/Other	1027	260.0	4.0 (3.7–4.2)	
Age groups				
30–39	31	5.3	5.8 (4.0–8.3)	1.5 (0.9–2.0)
40–49	94	19.5	4.8 (3.9–5.9)	1.2 (0.9–1.5)
50–59	245	54.9	4.5 (3.9–5.1)	1.1 (0.9–1.3)
60–69	446	111.3	4.0 (3.6–4.4)	Reference
70–79	216	70.1	3.1 (2.7–3.5)	0.8 (0.7–0.9)
Body sites of first melanoma				
Face	233	72.1	3.2 (2.8–3.7)	Reference
Scalp and neck	169	40.9	4.1 (3.5–4.8)	1.3 (1.0–1.6)
Trunk	82	16.0	5.1 (4.1–6.4)	1.6 (1.2–2.0)
Upper limbs	303	74.5	4.1 (3.6–4.6)	1.3 (1.1–1.5)
Lower limbs	245	57.7	4.2 (3.7–4.8)	1.3 (1.1–1.6)

The SIRs in male were 4.5 for invasive and 3.8 for in situ cohort while it was 4.8 in invasive and 4.2 in in situ melanomas of females (Table 5). The IRR in males was 10% lower in both cohorts compared to females which was not statistically significant (95% CI 0.8–1). The IRR of Māori was 120% higher significantly than that of European for invasive melanoma, but for in situ cohort there was no significant difference between ethnic groups (ratio 1, 95% CI 0.1–1.9). Subsequent invasive melanoma were more likely to appear in the younger age groups in both types of primary melanoma. The SIRs of primary melanoma in trunk was 5.8 for invasive and 5.1 for in situ melanomas. Therefore, patients with primary melanoma in trunk were more likely to be diagnosed with subsequent invasive melanoma than those with primary lesion on face for invasive cohort, but for in situ cohort, the IRR of primary lesions at any regions of the body was higher than that on face.

## 5 DISCUSSION

This study found that the cumulative incidence of subsequent invasive melanoma increased similarly for both primary invasive and in situ melanoma cohorts. The risk of subsequent invasive melanoma was marginally higher for primary invasive compared to in situ melanoma after adjustment for age, sex, ethnicity and body site of the initial lesion. While we found little difference in risk of subsequent invasive melanoma according to whether primary melanoma was invasive or in situ, we did find differences in the risk of this outcome according to other factors: sex, ethnicity, age groups and body sites of first melanoma. Risk was higher in men than women, in Europeans than Māori and in scalp and neck than face among primary lesion.

The 5‐year cumulative risk of subsequent melanoma observed in our study conducted in New Zealand, 2001–2017 (4.2% invasive, 3.8% in situ) is similar to that observed in a previous study conducted in Australia (3.8‐5.0% invasive, 3.6‐6.7% in situ),^30^ but higher than the findings of studies conducted in the Netherlands (1.8% invasive, 2.1% in situ)^31^ and the United States (1.2% invasive, 1.3% in situ).^32^ Likewise, the SIR of subsequent invasive melanoma in our study (4.6% invasive, 4.0% in situ) was similar to previous studies^27^, ^30^ in Queensland (SIRs = 5.4–8.2 invasive, 4.6–7.4 in situ). In the Dutch study,^31^ the SIRs (12.4 invasive, 15.4 in situ) was much higher than that identified in Australian studies. This much higher SIRs of subsequent invasive melanoma compared with the general population and the lower risk of cumulative incidence observed in Netherlands compared with our study and those in Queensland may be because the Netherlands^22^, ^39^ has lower background incidence rates of melanoma than in Australia^7^, ^8^, ^9^, ^10^ and in New Zealand.^11^, ^12^

Multiple factors influence the identification of subsequent invasive melanoma following primary melanoma including follow‐up surveillance.^40^ The aims of follow‐up have included identification of recurrence or new melanomas^41^, ^42^, ^43^ and also to provide psychosocial support and training for self‐examination.^43^ Follow‐up guidelines and schedules are not standardized and vary considerably between countries.^41^, ^43^, ^44^ A previous systematic review found that schedules varied according to country, physician speciality and stage of disease.^44^

Some guidelines do not mention^42^, ^45^ or do not include^46^ in situ melanoma as part of their advice for melanoma follow‐up, despite the fact that, as with our study, previously published studies^27^, ^30^, ^31^, ^32^, ^34^ have found that the risk of subsequent invasive melanoma is similar whether the primary lesion is in situ or invasive. In New Zealand, there are no differences in follow‐up surveillance after primary invasive or in situ melanoma.^43^ Follow‐up schedules depend on patient risk and needs, especially disease staging. Patients are trained to undertake self‐examination, but the responsibility for melanoma follow‐up remains with a lead health care professional (specialist, general practitioner, nurse practitioner or a combination) experienced in melanoma diagnosis and management.^43^

A recent study suggested follow‐up surveillance should be based on individual risk assessment in order to adequately balance potential benefits and costs (direct surveillance costs and patient harms).^40^ At the same time, results from a pilot of the MELanoma SELF surveillance (MEL‐SELF) randomized clinical trial^47^, ^48^ indicate that patient‐led surveillance for localized melanoma appears likely to be a safe, feasible and cost‐effective alternative to clinician‐led surveillance. Full results from the main MEL‐SELF trial will be available following completion of the trial in 2023.

This is the nation‐wide population‐based study that compared the subsequent risk of invasive melanoma between primary invasive and in situ melanoma patients. This study has considered key demographic characteristics (age, sex and ethnicity) as well as body site in estimating absolute and SIR of subsequent invasive melanoma among primary melanoma patients. One limitation of our ability to investigate subsequent invasive melanoma risk is that we were unable to assess the effect of other relevant factors (especially histopathological, phenotypical and genomic) as these data are not yet routinely included in the National Cancer Registry. This study was limited to the patients who are New Zealand residents and a limitation would be the failure to include international emigrants who were diagnosed with their first melanoma (either invasive or in situ) in New Zealand but had their subsequent melanoma diagnosed in other countries. However, in the relevant age ranges, permanent emigration from New Zealand is uncommon.^49^ In this study we focussed only on patients with their first primary melanoma (either invasive or in situ) who were diagnosed with a first subsequent invasive melanoma and all analyses were considered at patient‐level. The consideration of further subsequent lesions can be influenced by differences in follow‐up practices and in recording.

In conclusion, our study suggests that the risk of a subsequent invasive melanoma are similar for patients presenting with either a primary invasive or in situ melanoma, since the absolute risk and SIRs of subsequent invasive melanoma were similar in both cohorts. Thus follow‐up surveillance for new lesions should be similar, although patients with invasive melanoma require more frequent surveillance for recurrence.

## CONFLICT OF INTEREST

The authors declare no conflict of interest.

## ETHICS APPROVAL

This study is approved by the University of Auckland Human Participants Ethics Committee (UAHPEC) with Ref. 024010.

## AUTHOR CONTRIBUTIONS

**Thu Thu Win Myint:** Data curation; Formal analysis; Methodology; Software; Validation; Visualization; Writing – original draft; Writing – review & editing. **Vanessa Selak:** Methodology; Software; Supervision; Validation; Writing – review & editing. **Mark Elwood:** Conceptualization; Methodology; Supervision; Validation; Writing – review & editing.

## Supporting information

Supporting Information S1

## Data Availability

The data for this study were provided by the New Zealand Ministry of Health, and may be available to other researchers who meet data access requirements. Please contact data_enquiries@moh.govt.nz for further details on eligibility and data provision.
