# Supplementary material for: The risk of subsequent invasive melanoma after a primary in situ or invasive melanoma in a high incidence country (New Zealand)
Source: Skin Health Dis. 2022 May 13;3(2):e116. doi: 10.1002/ski2.116 (PMC10066759; doi:10.1002/ski2.116)
Supplement: Supplementary file 1 — Supporting Information S1 [file SKI2-3-e116-s001.docx]

Supporting information

****Appendix S1. Calculation of expected incidence of invasive melanoma****

For a specific patient, the expected rate of invasive melanoma in the first year follow-up, derived from national cancer registry data by the method below, is A.

The expected rate in the second year of follow-up is A(1+*i*), were *i* is the annual proportional change (APropC), which is sum of the APropC for one year of age, and the APropC for one year of time.

The cumulative rate R after F years of follow-up is

$$\sum_{f=1}^{f=F} A\left( 1+i \right){}^{(f-1)}$$

Regressions of ln (A) by age, and by calendar year, both in single years, show linear relationships within the range of data used. The expected rate in the first year of follow-up is calculated from the precise age of the patient and the calendar year, and the APropC per year of age and APropC per calendar year are derived from the regressions, shown in supplemental table 1.

The formula above is mathematically equivalent to

$$R={A (1+i)}^{n}+A \frac{{(1+i)}^{n}-1}{i}$$

which is used in financial work as the future value of an annuity (https://www.educba.com/future-value-of-an-annuity-formula/).

Table S1: Regressions of ln (rate) by age and by calendar time.

|  |  | NZ European | | Maori | |
| --- | --- | --- | --- | --- | --- |
| Regression of ln (A) by age | | Males | Females | Males | Females |
| intercept | 0.9737 | 2.2744 | 0.6780 | 0.3890 |  |
|  | slope, b | 0.0665 | 0.0416 | 0.0611 | 0.0458 |
|  | R2 | 0.996 | 0.988 | 0.901 | 0.972 |
|  | APropC per 1 year of age | 0.0688 | 0.0425 | 0.063 | 0.0469 |
|  |  |  |  |  |  |
| Regression of ln (A) by year | |  |  |  |  |
|  | slope, b | 0.0042 | –0.0004 | 0.022 | -0.0035 |
|  | R2 | 0.18 | 0.00 | 0.12 | 0.01 |
| APropC per 1 year of time | 0.0042 | –0.0004 | 0.0220 | -0.0035 |  |
|  |  |  |  |  |  |
|  |  |  |  |  |  |
| where *A* = incidence rate of invasive melanoma in general population. | | | |  |  |
| Regressions based on data for age range 30–74, for each ethnic group. | | | |  |  |
| APropC (on linear scale) = exp(b)-1 | |  |  |  |  |

Table S2. Relative risk ratio between the two types of primary melanoma

| Key characteristics | Primary melanoma type | Observed | Expected | O/E Ratio with 95% CIs | Ratio of primary invasive compared to in situ melanoma |
| --- | --- | --- | --- | --- | --- |
| **Sex** | | | | | |
| Female | Invasive | 522 | 108.9 | 4.8 (4.4- 5.2) | **1.2 (1.0- 1.3)** |
|  | In situ | 416 | 100.1 | 4.2 (3.8- 4.6) |  |
| Male | Invasive | 751 | 168.3 | 4.5 (4.1- 4.8) | **1.2 (1.0- 1.3)** |
|  | In situ | 616 | 161.1 | 3.8 (3.5- 4.1) |  |
| **Ethnicity** | | | | | |
| European | Invasive | 1259 | 275.8 | 4.6 (4.3- 4.8) | **1.2 (1.1- 1.3)** |
|  | In situ | 1027 | 260.0 | 4.0 (3.7- 4.2) |  |
| Maori | Invasive | 14 | 1.4 | 9.9 (5.4- 16.7) | **2.5 (1.0- 12.8)** |
|  | In situ | 5 | 1.3 | 3.9 (1.2- 9.3) |  |
| **Age groups** | | | | | |
| 30-39 | Invasive | 67 | 8.7 | 7.7 (6.0- 9.8) | 1.3 (0.9- 2.1) |
|  | In situ | 31 | 5.3 | 5.8 (4.0- 8.3) |  |
| 40-49 | Invasive | 157 | 27.4 | 5.7 (4.9- 6.7) | 1.2 (0.9- 1.6) |
|  | In situ | 94 | 19.5 | 4.8 (3.9- 5.9) |  |
| 50-59 | Invasive | 352 | 62.0 | 5.7 (5.1- 6.3) | **1.3 (1.1- 1.5)** |
|  | In situ | 245 | 54.9 | 4.5 (3.9- 5.1) |  |
| 60-69 | Invasive | 442 | 108.2 | 4.1 (3.7- 4.5) | 1.0 (0.9- 1.2) |
|  | In situ | 446 | 111.3 | 4.0 (3.6- 4.4) |  |
| 70-79 | Invasive | 255 | 71.0 | 3.6 (3.2- 4.1) | 1.2 (1.0- 1.4) |
|  | In situ | 216 | 70.1 | 3.1 (2.7- 3.5) |  |
| **Body sites of primary melanoma** | | | | | |
| Face | Invasive | 105 | 25.1 | 4.2 (3.4- 5.1) | **1.3 (1.0- 1.6)** |
|  | In situ | 233 | 72.1 | 3.2 (2.8- 3.7) |  |
| Scalp & neck | Invasive | 315 | 72.4 | 4.4 (3.9- 4.9) | 1.1 (0.9- 1.3) |
|  | In situ | 169 | 40.9 | 4.1 (3.5- 4.8) |  |
| Trunk | Invasive | 88 | 15.2 | 5.8 (4.6- 7.1) | 1.1 (0.8- 1.5) |
|  | In situ | 82 | 16.0 | 5.1 (4.1- 6.4) |  |
| Upper limbs | Invasive | 477 | 99.3 | 4.8 (4.4- 5.3) | **1.2 (1.0- 1.4)** |
|  | In situ | 303 | 74.5 | 4.1 (3.6- 4.6) |  |
| Lower limbs | Invasive | 288 | 65.2 | 4.4 (3.9- 5.0) | 1.0 (0.9- 1.2) |
|  | In situ | 245 | 57.7 | 4.2 (3.7- 4.8) |  |
